# A 31-plex panel for high-dimensional single-cell analysis of murine preclinical models of solid tumors by imaging mass cytometry

**DOI:** 10.3389/fimmu.2022.1011617

**Published:** 2023-01-19

**Authors:** Yaël Glasson, Laure-Agnès Chépeaux, Anne-Sophie Dumé, Philippe Jay, Nelly Pirot, Nathalie Bonnefoy, Henri-Alexandre Michaud

**Affiliations:** ^1^ Institut de Recherche en Cancérologie de Montpellier (IRCM), Univ Montpellier, Inserm, Institut régional du Cancer de Montpellier (ICM), Plateforme de Cytométrie et d’Imagerie de Masse, Montpellier, France; ^2^ Institut de Recherche en Cancérologie de Montpellier (IRCM), Univ Montpellier, Inserm, Institut régional du Cancer de Montpellier (ICM), Montpellier, France; ^3^ Institut de Génomique Fonctionnelle (IGF), University of Montpellier, Centre national de la recherche scientifique (CNRS), Inserm, Montpellier, France; ^4^ BioCampus Montpellier, Univ Montpellier, Centre national de la recherche scientifique (CNRS), Inserm, Réseau d’Histologie Expérimentale de Montpellier, Montpellier, France

**Keywords:** Imaging mass cytometry, preclinical mouse model, tumor microenvironment, immune signature, high dimensional multiplexing, cellular network

## Abstract

Currently, the study of resistance mechanisms and disease progression in cancer relies on the capacity to analyze tumors as a complex ecosystem of healthy and malignant cells. Therefore, one of the current challenges is to decipher the intra-tumor heterogeneity and especially the spatial distribution and interactions of the different cellular actors within the tumor. Preclinical mouse models are widely used to extend our understanding of the tumor microenvironment (TME). Such models are becoming more sophisticated and allow investigating questions that cannot be addressed in clinical studies. Indeed, besides studying the tumor cell interactions within their environment, mouse models allow evaluating the efficacy of new drugs and delivery approaches, treatment posology, and toxicity. Spatially resolved analyses of the intra-tumor heterogeneity require global approaches to identify and localize a large number of different cell types. For this purpose, imaging mass cytometry (IMC) is a major asset in the field of human immuno-oncology. However, the paucity of validated IMC panels to study TME in pre-clinical mouse models remains a critical obstacle to translational or basic research in oncology. Here, we validated a panel of 31 markers for studying at the single-cell level the TME and the immune landscape for discovering/characterizing cells with complex phenotypes and the interactions shaping the tumor ecosystem in mouse models.

## Introduction

Cancer progression and response to treatment are modulated by complex intrinsic and extrinsic biological mechanisms. The tumor microenvironment (TME) is composed of malignant and non-malignant cells that constantly interact forming a complex network. This ecosystem changes over time and space, shaping the tumor architecture and heterogeneity. Importantly, it has been established that the tumor cell composition dramatically affects the treatment response. In this context, the immune contexture of solid tumors has been extensively studied, especially since the development of immunotherapies against immune checkpoints. It has been suggested that the immune cell localization and functional states may predict the treatment response. For instance, the immunoscore, based on the density of CD3^+^ and CD8^+^ T cells in the tumor and its invasive margins, can reliably predict the recurrence risk in patients with colorectal cancer (1, 2). The spatial distribution of B cells and their network of interactions also are considered predictive markers. Depending on their localization and interactions, B cells display opposite role. When dispersed, B cells progressively acquire regulatory functions and are associated with poor prognosis. Conversely, when they are organized in tertiary lymphoid structures, their abundance is associated with anti-tumor functions and predicts good outcome to blockade of the programmed cell death-1 (PD-1) receptor and its ligand PD-L1 (3–6).This emphasizes the TME complexity and the need to better characterize cell interactions within the tumor.

Mouse preclinical models are widely used to extend our understanding of the TME. Such models have become more and more sophisticated, from mice grafted with tumor cells to organ specific-induced tumors or genetically engineered models (7–9). As the immune system physiology is similar in mice and humans, preclinical mouse models can be used to decipher the complex relationships between cancer cells and immune cells and to extrapolate findings to humans (10, 11). For instance, mouse models led to many key discoveries in cancer treatment, including the immune checkpoint-based immunotherapies (12–14). P.S. Hedge and D.S. Chen identified the development and use of preclinical models as a major challenge in cancer immunotherapy to translate basic research findings to the clinic and also clinical findings back to preclinical models to better characterize the tumor biology (15). Besides studying the tumor cell interactions with their environment, mouse models allow assessing the efficacy of new drugs and delivery approaches, treatment posology and toxicity, and their impact on the TME, which cannot be done using human samples (16).

Single-cell high dimensional imaging mass cytometry (IMC) has been developed to investigate the tumor cell architecture and interactions. IMC combines immunohistochemistry and mass cytometry and can simultaneously detect ~40 single markers in situ. Tissue sections are labeled with metal-tagged antibodies. Then, selected regions of interest (ROI) are ablated with a UV laser (resolution of 1µm^2^) and the metal tags are detected by time-of-flight mass spectrometry. This allows overcoming the limitations frequently observed with fluorescent tags, particularly spectral overlapping and auto-fluorescence. Cell segmentation from multiplexed images is used to generate a single-cell file (.fcs or.csv) in which the mean expression of each marker in each cell and its spatial coordinates (X and Y) on the slide are associated.

Several studies have highlighted IMC value for cancer research. Indeed, IMC has allowed better describing the TME cell heterogeneity and networks, discovering novel phenotypes, and precisely characterizing the treatment response in patients (17). Although many antibodies have been validated for high-plex imaging of human tissue samples, none or very few antibodies have been evaluated for IMC analysis of formalin-fixed paraffin-embedded (FFPE) mouse tissue samples. In this work, we validated an original panel of 31 antibodies for the IMC analysis of different FFPE mouse samples, including lymphoid organs and tumor models (e.g. melanoma and intestinal tumors). As previously reported for human and non-primate antibody panels (18–21), this panel allows characterizing the TME architecture and the immune contexture. We also demonstrated that this antibody panel and dedicated analyses can be used to identify cells with complex or atypical phenotypes, to localize them, and to deduce their network within the TME.

## Results

### Antibody validation and complex tissue architecture visualization

We selected the 31 markers to identify immune and non-immune cells and their different functional states (**Table 1**). As the IMC workflow includes a single staining step, we needed to confirm that the same antigen retrieval method was compatible with all 31 antibodies. Therefore, we assessed whether the antigen retrieval step commonly used for conventional immunofluorescence worked with all antibodies in the relevant tissues (**Sup Figure 1**). After validation of the antigen retrieval step, we conjugated all 31 antibodies with metal isotopes and tested them by IMC using a tissue microarray (TMA) representative of eight mouse normal tissues (**Sup Figure 2**). The tested panel included markers to identify macrophages (CD11b, CD68, and IBA-1), dendritic cells (CD11c), plasmacytoid cells (CD11c, B220), granulocytes (MPO), other myeloid cells (CD11b), B cells (CD19, B220), CD4^+^ and CD8^+^ T cells (CD3, CD4, and CD8), and natural killer (NK) cells (NCR-1). It also included functional markers to better define the immune cell infiltrate: M1/M2 macrophage balance (CD80), cytotoxicity (granzyme-B), T helper type 2 (Th2) cells (GATA-3), regulatory T cells (FOXP3), T helper type 17 (Th17) cells (RORγt), activation/antigen presentation (MHC-II), apoptosis (cleaved caspase-3), proliferation (Ki-67), residency (CD103), and activation/exhaustion (CD39). To study the non-immune compartment, the panel included phenotypic markers of smooth muscle cells (αSMA), lymphatic vessels (LYVE-1), high endothelial venules (PNAd), epithelial cells (pan-cytokeratin and E-cadherin), fibroblasts (vimentin), endothelial cells (CD31), cell adhesion (β-catenin), and extracellular matrix (pan-actin and collagen-I).

**Table 1:** Antibody Panel.

Target	Clone	Supplier	Metal	Dilution	Function
αSMA	1A4	SantaCruz	^110^Cd	1/1000	Smooth muscle cells
IBA1	EPR16588	Abcam	^141^Pr	1/500	Macrophages
CD19	6OMP31	Fluidigm	^142^Nd	1/800	B cells
Vimentin	D21H3	Fluidigm	^143^Nd	1/1000	Fibroblasts
B220	RA3-6B2	Fluidigm	^144^Nd	1/800	B cells
NCR1	EPR23097-35	Abcam	^145^Nd	1/400	Natural killer cells
E-cadherin	24E10	Cell Signaling	^146^Nd	1/100	Epithelial cells
Granzyme B	D2H2F	Cell Signaling	^147^Sm	1/100	Cytotoxicity
Pan-Cytokeratin	AE1/AE3	SantaCruz	^148^Nd	1/200	Epithelial cells
CD11b	EPR1344	Fluidigm	^149^Sm	1/100	Myeloid cells
CD31	EPR17259	Abcam	^151^Eu	1/200	Endothelial cells
CD3	CD3-12	Abcam	^152^Sm	1/400	T cells
CD8a	EPR21769	Abcam	^153^Eu	1/400	CD8^+^ T cells
CD11c	D1V9Y	Cell Signaling	^154^Sm	1/400	Dendritic cells
RORγt	EPR20006	Abcam	^155^Gd	1/200	Th17 cells
CD80	Polyclonal	Abcam	^156^Gd	1/400	M1/M2 balance
FOXP3	D6O8R	Cell Signaling	^158^Gd	1/400	Regulatory T cells
CD68	polyclonal	Proteintech	^159^Tb	1/1000	Macrophages
PNAd	MECA-79	Biolegend/Ozyme	^162^Dy	1/1000	High Endothelial Venule
CD4	BLR167J	Bethyl	^163^Dy	1/100	CD4^+^ T cells
MPO	EPR20257	Abcam	^164^Dy	1/2000	Neutrophils
β-catenin	D13A1	Fluidigm	^165^Ho	1/400	EMT
MHC-II	REA813	Miltenyi	^166^Er	1/200	Antigen presentation
GATA-3	EPR16651	Abcam	^167^Er	1/400	Th2 cells
Ki-67	B56	Fluidigm	^168^Er	1/500	Proliferation
Collagen-I	polyclonal	Fluidigm	^169^Tm	1/500	Extracellular matrix
LYVE-1	polyclonal	Abcam	^171^Er	1/100	Lymphatic vessels
Caspase-3	5AE1	Fluidigm	^172^Yb	1/200	Apoptosis
CD103	EPR22590-27	Abcam	^174^Yb	1/200	Residency
Pan-actin	D18C11	Fluidigm	^175^Lu	1/200	Extracellular matrix
CD39	EPR20627	Abcam	^176^Yb	1/100	Immune Checkpoint
Iridium	/	Fluidigm	^191/193^Ir	1/400	Nucleus

**Figure 1:**
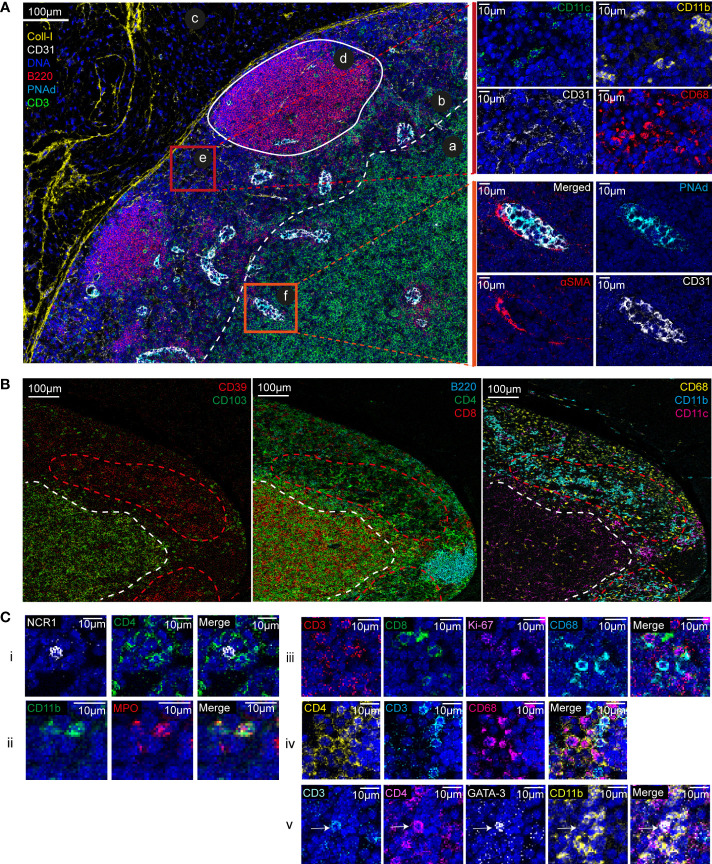
Lymph node anatomy, cell composition, and cell localization revealed by IMC. **(A)** Multiplexed pseudo-colored images showing the lymph node architecture: (a) cortex, (b) paracortex, (c) capsule, (d) germinal center, (e) cortical sinus, and (f) high endothelial venule. The white dashed line separates the paracortex from the cortex. Upper zoom (red square): cell composition of the cortical sinus (e) assessed by CD11c, CD11b, CD31 and CD68 signal analysis. Insets (orange square): high endothelial venule identified by PNAd, CD31, and αSMA expression. **(B)** Multiplexed pseudo-colored images showing the CD103- and CD39-positive areas. Green and red dashed lines delimit the CD103- and CD39-positive areas, respectively. Lymphoid areas are identified by B220, CD4, and CD8 expression. Myeloid areas are identified by CD11b, CD11c and CD68 expression. **(C)** Single-cell analysis and cell interactions: (i) NK cell (NCR-1^+^), (ii) neutrophil (CD11b^+^MPO^+^), (iii) two proliferating CD8+ T cells (CD8^+^Ki-67^+^) and a macrophage (CD68^+^), (iv) discrimination between CD4^+^ T cells (CD3^+^CD4^+^CD68^-^) and CD4^+^ macrophages (CD68^+^CD4^+^CD3^-^), and (v) a Th2 T cell (CD3^+^CD4^+^GATA-3^+^) in contact with a myeloid cells (CD11b^+^).

Using IMC and the set of cell markers we could thoroughly investigate the anatomical and cellular organization of lymph node tissue (**Figure 1**). By analyzing the obtained multiplexed images, we identified the typical lymph node areas, such as the B cell-rich cortical area and the T cell-rich paracortical area (**Figure 1A**, ab). In the cortical area, two germinal centers were visible (**Figure 1A**, d) with high density of B cells, a characteristic of secondary follicles. At the periphery, we identified the capsule, characterized by the presence of collagen-I (**Figure 1A**, c). We also detected high endothelial venules, specialized blood vessels for lymphocyte recruitment in lymph nodes, on the basis of the presence of PNAd-expressing endothelial cells (CD31^+^) (**Figure 1A**, f). Between germinal centers, we identified a cortical sinus (**Figure 1A**, e), characterized by the presence of small blood vessels (CD31^+^), macrophages (CD68^+^), dendritic cells (CD11c^+^) and other myeloid cells (CD11b^+^). Functional markers, such as CD103 (integrin alpha-E beta-7) and the ectonucleotidase CD39, helped us to better define the lymph node area. Although poorly described in the literature, we observed that CD103 expression was spatially defined and associated with the T-cell area (CD4^+^, CD8^+^). Similarly, and in accordance with our previous work, CD39 expression was localized in an area rich in myeloid cells (CD11b^+^) that constitutively express this enzyme (**Figure 1B**) (22).

We also identified and localized cells that are poorly present in lymph nodes, such as NK cells (NCR-1^+^) and neutrophils (CD11b^+^MPO^+^) (**Figure 1C**, i and ii). The 1µm resolution allowed visualizing contacts between immune cells. For instance, we identified a proliferating (Ki-67^+^) CD8^+^ T cell in contact with a macrophage (CD68^+^) (**Figure 1C**, iii). The high-plex IMC capacity improved tissue phenotyping accuracy. For instance, we could determine whether CD4 was expressed by a T cell or a macrophage (**Figure 1C**, iv and v). We also detected complex phenotypes (**Figure 1C**, v), such as a Th2 cell (CD3^+^CD4^+^GATA3^+^) in contact with several myeloid cells (CD11b^+^).

### Immune cell distribution in a preclinical mouse model of melanoma

**Figure 2:**
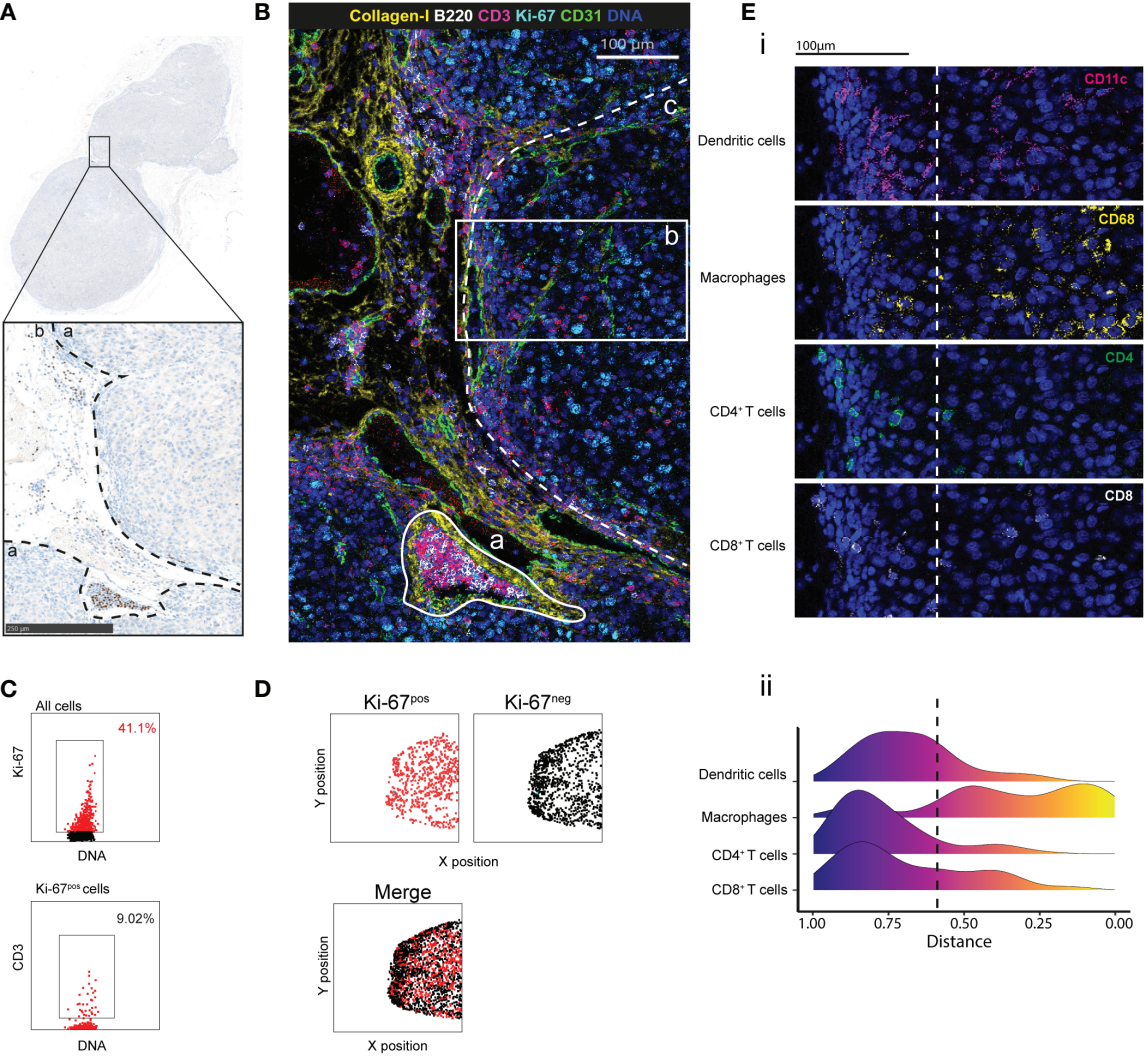
Cell distribution and tumor microenvironment characterization by IMC. **(A)** IHC staining with PAX-5 (brown) and hematoxylin (blue) of a region of interest (ROI) in a B16-K1 melanoma cell graft tissue section. The black dashed lines delimitate the tumor (a) and the peritumoral (b) areas. **(B)** Pseudo-colored image of the ROI. Overlaid signals of collagen I (extracellular matrix), B220 (B cells), CD3 (T cells), CD31 (endothelial cells), Ki-67 (proliferation) and DNA (nucleus). The white lines delimit: (a) a lymphatic vessel, and (b) a tumor area from the tumor core to the periphery. The white dashed line demarcates the main tumor area (c). **(C)** Proliferating cells and T cells identified by manual gating. (3). **(D)** Distribution of Ki-67-positive (red) and Ki-67-negative (black) cells in the main tumor area, assessed as in **(C)**. **(E)** (i) T-cell exclusion in the tumor core within the tumor area (2) in **(B)** confirmed by the CD11c (dendritic cells), CD68 (macrophages), CD4 (CD4+ T cells) and CD8 (CD8+ T cells) signals. (ii) Distribution of dendritic cells, macrophages, CD4+ and CD8+ T cells in the tumor. For each cell, its distance from the tumor core was calculated. The color scale indicates the distance from the core. The dashed line represents the border between tumor core and periphery.

**Figure 3:**
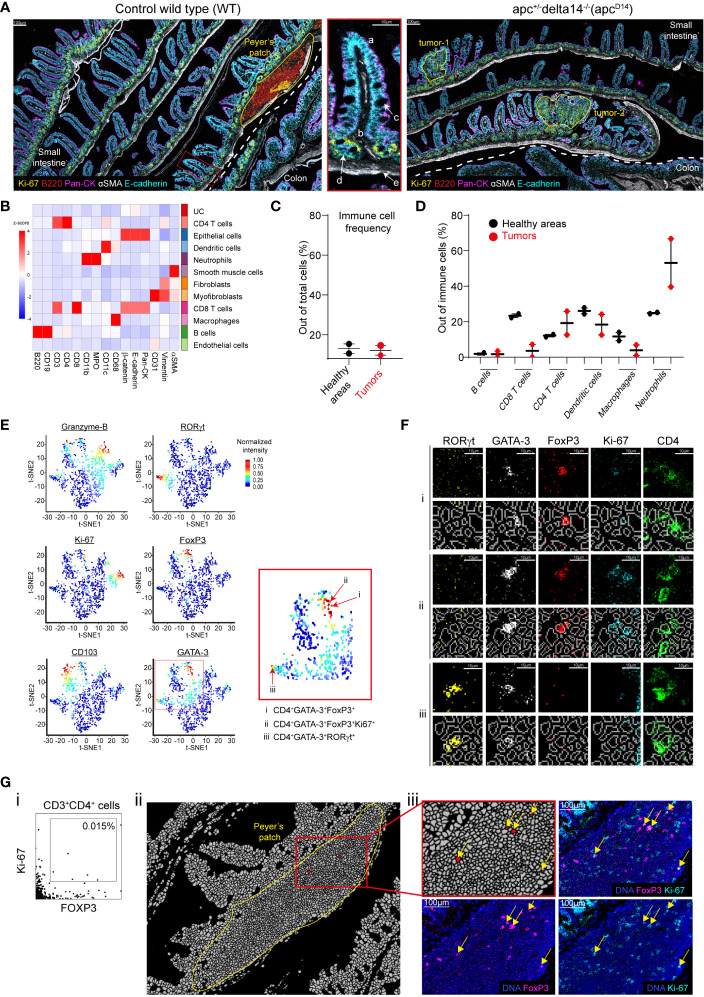
Single-cell analysis and validation. **(A)** Pseudo-colored images of gut sections from a WT and *Apc* ^Δ14+^ mouse. The dashed white line separates small intestine and colon. The areas of interest (the Peyer’s patch and two tumors) are delineated by yellow lines. The zoomed inset (middle panel) represents a villus (a) with the lamina propria (b), goblet cells (c), crypts (d), and muscularis mucosae (e) in the WT small intestine. **(B)** Heatmap showing the z-score of the expression of the indicated markers in the 11 cell clusters generated by Phenograph after cell segmentation of the WT and *Apc* ^Δ14+^ images. UC: unidentified cluster. **(C)** Immune cell frequencies in the two healthy zones H*^Apc^* and H^WT^ (black dots), and in the two tumors (red dots). Immune cells were defined using the clusters identified in **(B)**. The Peyer’s patch was excluded from the analysis. **(D)** Percentage of each immune cell cluster identified in **(B)** among all immune cells present in two healthy area H*^Apc^* and H^WT^ (black dots) and the two tumors (red dots). **(E)** t-SNE representation of the expression of functional markers in the CD4^+^ T-cell cluster identified in **(B)**. Red arrows in the red square shows CD4^+^ T cells expressing multiple functional markers. **(F)** Validation of the functional marker expression on cells identified in **(E)**: i: CD4^+^FOXP3^+^GATA-3^+^, ii: CD4^+^FOXP3^+^GATA-3^+^Ki-67^+^. iii: CD4^+^FOXP3^+^RORγt^+^ cells. For each cell phenotype, upper lines represent single marker expression and bottom lines show the single marker expression combined with the segmentation mask. **(G)** (i) Manual gating identified CD3^+^CD4^+^FOXP3^+^Ki-67^+^ cells. (ii) Localization of the cells identified in (**G**, i). The image corresponds to the segmentation mask of the Peyer’s patch and the red cluster corresponds to CD3^+^CD4^+^FOXP3^+^Ki-67^+^ cells. (iii) Validation of the expression of FOXP3 and Ki-67 in the cells identified in (**G**, i) using the raw images.

Next, we tested our antibody panel using tumor tissue samples from C57Bl/6 mice grafted with the B16-K1 melanoma cell line (23, 24). This preclinical model has been used to investigate the melanoma immune contexture and the effects of immunotherapies (25). We identified the tumor and peritumoral areas in FFPE tumor sections stained with hematoxylin to detect the nucleus (**Figure 2A**). After IMC, we could detect the different tissue components, such as large vascular structures (CD31^+^) that were surrounded by collagen-I and a tubular structure that was reminiscent of a lymphatic vessel full of B (B220^+^) and T (CD3^+^) cells in the peritumoral area (**Figure 3B**). Analysis of the immune contexture showed that T cells accumulated at the tumor periphery and that very few T cells efficiently infiltrated the tumor core. Ki-67 expression distribution highlighted an active tumor nodule with many more Ki-67-expressing cells compared with the peritumoral area (**Figure 2B**). After cell segmentation, we observed that about 41% of cells within the tumor were proliferating (of which only 9% were T cells) (**Figure 2C**) and were homogenously distributed in the tumor area. Conversely, Ki-67-negative cells segregated at the periphery (**Figure 2D**). By calculating the distance of each cell type from the tumor center, we objectively demonstrated the exclusion of T and dendritic cells from the tumor core in this tumor node (**Figure 2E**). Altogether, this showed that this antibody panel can be used to comprehensively describe the main compartments and specific cell phenotypes also in a mouse model of a solid tumor. Furthermore, objective parameters could be extracted from the single-cell data to support the descriptive observation, for instance the T cell exclusion.

### Comparative single-cell analysis of healthy, lymphoid, and tumor tissue samples from the intestine

We then analyzed and compared by IMC intestine tissue sections from wild type (WT; healthy tissue) and *Apc* ^Δ14+^ C57Bl/6 mice that spontaneously develop intestinal tumors (26). The pan-cytokeratin (epithelial cells), αSMA (smooth muscle cells) and E-cadherin (epithelial cells) markers allowed identifying the characteristic intestine structures (**Figure 3A**). In WT intestine sections, we identified a Peyer’s patch on the basis of the high density of B cells (B220), among which some were proliferating (Ki-67^+^), and of T cells. IMC resolution allowed detecting the muscularis mucosae (αSMA^+^), proliferating epithelial cells in the crypts (E-cadherin^+^, Ki-67^+^), the lamina propria and villi, and mucin-secreting goblet cells (E-cadherin^+^) with a large nucleus that displayed apical polarized cytokeratin expression. We could distinguish small intestine and colon on the basis of the form and size of their villi (**Figure 3A**). The pathologist identified two tumor areas in the *Apc^Δ14+^* intestine samples (**Figure 3A**). Overall, we could describe five distinct areas: two tumor areas (tumor-1 and tumor-2) in the *Apc^Δ14^* intestine sections, the Peyer’s patch in WT intestine sections, and two healthy areas, one in the WT (H^WT^) and one in the *Apc^Δ14+^* (H*^Apc^*) sections (i.e. the whole tissue minus the Peyer’s patch or minus the two tumors, respectively).

To fully exploit the generated data, we performed a cellular segmentation of the two images that corresponded to the WT and *Apc^Δ14+^* intestine sections followed by unbiased cell clustering. Cell clustering identified eleven cell clusters: B cells, CD4^+^ T cells, CD8^+^ T cells, macrophages, dendritic cells, neutrophils, endothelial cells, epithelial cells, smooth muscle cells, myofibroblasts, and fibroblasts (**Figure 3B**). We then compared the frequency of the immune cell types identified in each area. The mean percentages of immune cells were similar in the two healthy areas (H^WT^ and H*^Apc^*) and in the two tumor areas (13% and 12%, respectively) (**Figure 3C**), but not their composition. Indeed, the image analysis suggested a decrease of CD8^+^ T cells in the two tumor areas (**Sup Figure 3**). Single-cell analysis confirmed that the proportion of CD8^+^ T cells was lower in the two tumors than in the two healthy areas (23% versus 4%). We then found that compared with the healthy tissues, the two tumor areas were enriched in neutrophils (25% versus 53%), whereas the percentage of macrophages was decreased (12% versus 4%) (**Figure 3D**). The concordance between raw marker expression and analysis of the post-segmentation data validated the segmentation process.

In the intestine, CD4^+^ T cell composition is particularly heterogeneous, including FOXP3^+^ regulatory, Th2 and Th17 T cells (27, 28). Therefore, we checked whether our antibody panel could detect cells with complex phenotypes. To this aim, we used t-distribution Stochastic Neighborhood Embedding (t-SNE) for dimensionality reduction of the functional marker expression data for the CD4^+^ cluster (**Figure 3E**). In the t-SNE plots, cells are distributed according to their expression profile. In this way, we could identify regulatory, Th2, and Th17 CD4^+^ T cells on the basis of their FOXP3, GATA-3 and RORγt expression profile. Moreover, we could discriminate between proliferating and resident CD4^+^ T cells on the basis of granzyme-B, Ki-67 and CD103 expression, respectively (**Figure 3E**). We also identified CD4^+^ T cells that expressed different marker combinations: FOXP3 and GATA-3 (**Figure 3E**, i), FOXP3, GATA-3 and Ki-67 (**Figure 3E**, ii), or RORγt and GATA-3 (**Figure 3E**, iii). To validate these observations, we localized these cells on the image and confirmed the expression of each marker (**Figure 3F**).

To assess whether we could detect also rare events, we looked for CD3^+^CD4^+^FOXP3^+^Ki-67^+^ T cells. By manual gating, we identified few cells that displayed this phenotype (i.e. 0.015% of all cells) (**Figure 3G**, i). To validate this finding, we first localized these cells and found that they were restricted to the Peyer’s patch (**Figure 3G**, ii). We then confirmed the expression of both FOXP3 and Ki-67 in CD4^+^ T cells (**Figure 3G**, iii).

Altogether, these results validated both the segmentation process and the possibility to identify rare cells.

**Figure 4:**
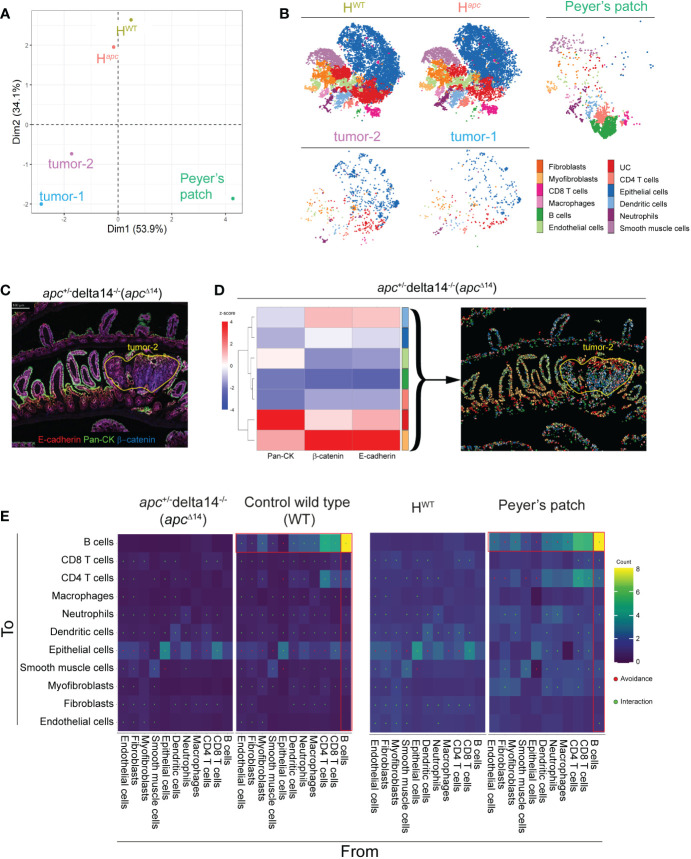
Comparison of cell heterogeneity and interactions in healthy and tumor intestine samples. **(A)** Principal component analysis of the five zones of interests based on the abundance of the clusters identified in (**Figure 3B**). H^WT^: whole WT tissue sample minus the Peyer’s patch; H*^Apc^*: whole *Apc* ^Δ14+^ tissue sample minus the two tumor nodes identified by a pathologist. **(B)** Visualization of high-dimensional images using t-SNE and based on marker expression. Each color corresponds to one of the clusters identified in (**Figure 3B**). **(C)** Pseudo-colored image showing the signal intensity for E-cadherin, β-catenin and pan-cytokeratin in the *Apc* ^Δ14+^ intestine tissue sample. **(D)** Heatmap showing the z-score of the marker expression in the sub-clusters generated by Phenograph based on E-cadherin, β-catenin and pan-cytokeratin expression from the epithelial cluster identified in (**Figure 3B**) and localized in the *Apc* ^Δ14+^ intestine tissue section. Each color identifies an individual cluster. **(E)** Heatmap in which dots indicate significant interaction or avoidance between the indicated clusters. Red squares indicate interactions (green) or avoidance (red) of B cells with the other clusters.

We then used principal component analysis (PCA) to determine whether the cell cluster frequencies were different in the two tumors, Peyer’s patch, and two healthy areas. PCA clustered the two tumors together, far away from the two healthy areas. As expected, the Peyer’s patch, mostly composed of immune cells, was separated from the other two tissue types (**Figure 4A**). This suggests a structural similarity of the two tumor areas and also of the two healthy areas, as indicated also by the high-dimensional visualization of each area by t-SNE (**Figure 4B**).

Multiplexed images also suggested different E-cadherin, β-catenin and cytokeratin expression patterns in function of the epithelial cell localization (**Figure 4C**). For instance, pan-cytokeratin expression (green) was mainly in apical enterocytes, whereas β-catenin (blue) expression was increased in the tumor areas. To investigate the epithelial cell heterogeneity, we performed sub-clustering based on the expression of E-cadherin, β-catenin and pan-cytokeratin, and identified seven clusters of epithelial cells. As expected, in the normal adjacent tissue, epithelial cell clusters were well organized and associated with the crypts or the villi. Conversely, in the two tumor areas, epithelial clusters were mixed, revealing an anarchic proliferation, as illustrated for tumor-2 in **Figure 4D**.

The Hyperion Imaging System offers the opportunity to reconstitute *in situ* the cell interaction network and to identify cell communities. Therefore, we assessed whether the antibody panel could identify specific structures based on cell-cell interactions. For this purpose, we evaluated the contacts between each cell cluster in the WT and *Apc^Δ14+^* samples. The cell networks were very similar in the two ecosystems (healthy tissues and tumors); however, we identified a pattern that mainly involved B-cell interactions in the WT tissue sample (**Figure 4E**, left panels). As the Peyer’s patch is particularly rich in B cells, we then split the WT sample into healthy area (H^WT^; minus the Peyer’s patch) and Peyer’s patch and compared their cluster interaction signatures. The results (**Figure 4E**, right panels) indicated that the previously observed difference between *Apc^Δ14+^* and WT samples was explained by the presence of the Peyer’s patch in the WT sample, and validated the possibility of studying cell-cell interactions with our antibody panel.

## Discussion

In the present work, we validated a 31-antibody panel suitable for IMC analysis of FFPE mouse samples. We tested the antibody panel using one lymph node sample and tumor samples isolated from the B16-K1 and the *Apc^Δ14+^* mouse models. We chose a lymph node to validate a large series of antibodies against immune markers because of their specific cell composition. Lymph nodes are the place where the immune response initiates and their analysis is crucial to decipher the immune mechanisms associated with a pathological state, such as the development of the anti-tumor response. *Apc^Δ14+^* mice that develop spontaneous intestinal tumors allowed comparing tumor and adjacent healthy tissues. The multiplexed images generated by IMC allowed us to characterize the tissue architecture, to identify small components (blood vessels, high endothelial venules, lymphoid structures), and to describe their cell composition. In tumors from the B16-K1 mouse melanoma model, IMC revealed that T cells segregate at the periphery and macrophages infiltrate the tumor core, a typical feature of immune-excluded tumors.

The cell segmentation brought additional information. This crucial step has to be optimized to avoid artefacts. For instance, aberrant phenotypes, such as CD3^+^CD19^+^ cells, may be detected due to miss-assignment of signals coming from two strongly interacting cells (29). To limit this issue, we tested different boundary adjustments and used the frequency of B220^+^CD3^+^ cells as a segmentation quality index (**Sup Figure 4**). In this report, we used the post-segmentation data to validate our antibody panel by detecting the expected complex phenotypes, such as proliferating cells or regulatory T cells. On the other hand, we validated the single-cell analysis by assessing the signal intensity in raw data. Single-cell data allow transforming subjective observations into quantifiable parameters, such as cell density or the distance between immune cells and tumor core. Using the post-segmentation data, we identified and localized cells with complex phenotypes, thus revealing the strong CD4^+^ T cell heterogeneity. It is known that Th17 and Th2 CD4^+^ T cells are present in the intestine. Unlike the Th1 and Th2 subsets that are considered definitive, the Th17 and regulatory T-cell subsets do not represent stably differentiated cells and retain some plasticity (30). This may explain the detection of rare and transient phenotypes, such as CD3^+^CD4^+^FOXP3^+^ cells that express GATA-3 or RORγt. We also identified rare events, such as CD4^+^FOXP3^+^Ki-67^+^ T cells. In mice, FOXP3 is expressed in regulatory T cells and in naive CD4^+^ T cells stimulated through their TCR in the presence of TGF-β (31). The specific localization of this cell cluster in the Peyer’s patch suggests the presence of newly activated CD4^+^ T cells rather than the presence of regulatory T cells. Unsupervised approaches allow investigating the heterogeneity in a cell population, a key question in oncology (32). In *Apc^Δ14+^* tumor tissue samples, clustering identified epithelial cell clones with different epithelial marker expression profiles. Their spatial distribution illustrated the loss of cell organization. Investigating cell heterogeneity, by associating clustering and spatial approaches, might help to determine the tumor cell origin and to better understand the transformation steps. Tissue modeling addresses interactions or avoidances between cell clusters. This approach has been used in many cancer studies (29). Here, we could specifically identify the Peyer’s patch thanks to the many significant interactions of B cells with other cell types.

Beside IMC, other technologies based on sequential staining offer the possibility of multiplexed imaging, for instance PhenoCycler (previously known as CODEX) (33) and MACSima (34). One limitation of sequential immunostaining is that it may modify the epitope affinity and damage the tissue architecture. Tissue auto-fluorescence also may limit the detection of a weakly expressed epitope. IMC overcomes the limitations of conventional immunofluorescence-based multiplex imaging and has been used in many studies based on human samples (C. C. 17, 35, 36). In the context of preclinical models, IMC has been used to study frozen mouse tissues that do not require tissue embedding, rehydration and antigen retrieval and that therefore, can be stained with antibodies validated for mass cytometry (37, 38). However, frozen sections are usually thick (6-12 µm) and this limits cell segmentation due to cell overlap. Furthermore, the absence of fixation may lead to the loss of the tissue morphology. FFPE tissue sections may overcome these limitations. In few studies, FFPE mouse samples have been analyzed by IMC, but with smaller panels (39; H.-C. 40–42). For instance, Peran et al., used a 7-antibody panel to investigate the interactions between cancer-associated fibroblasts and tumor cells. Gheiratmand et al. used a 8-antibody panel in a proof-of-concept study to validate the use of plastic slides and to generate 3D images. Lotsberg et al., recently described a 18-antibody panel without immune markers to study FFPE mouse spheroids (43). Our high-plex antibody panel that includes immune markers suitable for FFPE mouse samples will be useful for studies based on preclinical mouse models.

In conclusion, in this work we validated a 31-antibody panel to investigate the TME in mouse preclinical models. Our panel was designed to identify the main immune cell populations and also stromal and tumoral cells, and is also suitable for single-cell downstream analysis. IMC is in constant progress, and more metals can be added for antibody tagging. Thanks to the low or absent spectral overlap, it will be easy to add in-house validated and conjugated antibodies or to replace one antibody in our panel.

## Materials and methods

### Tissue samples

Unless specified, tissues samples were from C57BL/6 mice. No mouse was sacrificed for this study. Only unused mouse FFPE tissue blocks from previous studies were used. The TMA containing eight mouse tissues (spleen, thymus, lymph node, liver, lung, muscle, lung tumor, MCA-205 fibrosarcoma cell graft) was generated by the Imaging and Mass Cytometry platform, IRCM, Montpellier, France. Lymph node and B16-K1 mouse melanoma cell graft samples were from Dr Bonnefoy’s team. Intestine tissue samples from WT and *Apc^Δ14/+^* mice were from Dr Jay’s laboratory (26).

### Antibodies and metal conjugation

The anti-αSMA antibody was conjugated to the cadmium-110 (^110^Cd) isotope with the Maxpar^®^ MPC9 Antibody Labeling Kit. All other antibodies were labeled using the Maxpar^®^ X8 Antibody Labeling Kit according to the manufacturer’s instructions (PRD002 Rev 14, Fluidigm, Standard Biotools).

### Imaging mass cytometry: Tissue labeling

After deparaffinization and antigen retrieval using Dako Target Retrieval Solution at pH 9 (S236784-2, Agilent technologies) in a water bath (96°C for 30 min), 3µm tissue sections were encircled with a Dako Pen, incubated with Superblock™ (37515, ThermoFisher Scientific) at room temperature (RT) for 45 min, and then with FcR Blocking Reagent (130-092-575, Miltenyi) at RT for 1h. After three washes (8 min/each) in PBS/0.2% Triton X-100 (PBS-T), metal-tagged antibodies (list in **Table 1**) were diluted in PBS/1% BSA buffer. After incubation with the primary antibodies at 4°C overnight, sections were washed in PBS-T three times (8 min/each) and nuclei were stained with iridium (1:400 in PBS; Fluidigm, Standard Biotools), a DNA Intercalator, for 30 min at RT. Sections were washed in PBS for 5 min, then in distilled water for 5 min, and dried at RT for 30 min.

### Imaging mass cytometry: Data acquisition

Images were acquired with the Hyperion Imaging System (Fluidigm, Standard Biotools) according to the manufacturer’s instructions. After choosing the ROI in the section, the ROI was ablated with a UV laser at 200Hz. Data were exported as MCD files and visualized using the Fluidigm MCD™ viewer 1.0.560.6. The minimum and maximum thresholds were adapted for each marker and for each tissue for optimal visualization. Gamma was set to 1.

### Data analysis

Cell segmentation. Cell segmentation was done with the flexible multiplexed image segmentation pipeline based on pixel classification developed by Zanotelli et al. (44). Briefly, pixel classification and training were performed with Ilastik v1.3.3 to generate a probability. Then, a segmentation mask was created from the probability map using CellProfiler v4.1.3. For each cell identified, the mean intensity of each marker and the spatial coordinates were associated with the computeFeatures function in the R EBImage package (45) and exported in.fcs format using the R package flowCore (46). Single-cell marker expression signals were summarized using the mean pixel values for each channel. Single-cell data were censored at the 99th percentile to remove outliers. Identification of zones of interest. Tissue architecture and features were identified by a pathologist. For intestine section comparison, seven fcs file were generated: two files concerned the whole tissue sample (WT intestine and *Apc* ^Δ14/+^ intestine with tumors) and five files concerned the Peyer’s patch, the two intestinal tumors, and the two healthy zones (H^WT^ represents the whole healthy tissue minus the Peyer’s patch, and H*^Apc^* represents the whole *Apc* ^Δ14/+^ tissue minus the two tumor areas). Cell clustering. The Phenograph algorithm in R was used to analyze the dataset after the 99th percentile normalization to eliminate outliers and minimize background noise. The αSMA, B220, CD19, β-catenin, CD3, CD4, CD8, CD11b, CD11c, CD31, CD68, E-cadherin, MPO, pan-cytokeratin, and vimentin signals were used for cell clustering with a k-value set at 60. To further analyze CD4^+^ T cells, cells were sub-clustered using CD103, FOXP3, GATA-3, granzyme-B, Ki-67 and RORγt expression with a k value set at 30. Epithelial cells were sub-clustered using pan-cytokeratin, β-catenin, and E-cadherin with a k-value set at 1000. Heatmaps. Heatmaps were generated using the z-score for each marker. Hierarchical clustering was performed using Euclidean distances and according to the Ward’s linkage method in the R package pheatmap (47). High-dimensional data visualization in 2D maps. The Barnes-Hut t-SNE method was used to generate 2D maps after concatenation and normalization to the 99th percentile of all areas with perplexity = 30, initial dimensions = 110, and theta = 0.5. Principal Components Analysis. The frequencies of each cluster per zone were retrieved with the FlowJo software and analyzed with the PCA method proposed by FactoMineR (48). Distance. Each cell was assigned a distance value from a point arbitrarily defined as the tumor center using the following equation: (x-a)² ^+^(y-b)²=r, where a and b are the coordinates of the ROI center, x and y the cell position, and r the circle radius. Data were represented using the ggplot2 package (49). Cell-cell interactions. Cell-cell interactions were determined and visualized using the R packages imcRtools (50) and cytomapper (51), respectively. A permutation test was performed to define the interactions (positive or negative) with neighboring cells. Cells were considered as neighbors if the distance between them was <12 µm, and then counted using the Histocat expansion graph method of the buildSpatialGraph function that defines how many neighbors a cell of cluster A has with cluster B (given that it has at least one neighbor of type B). The default number of permutations was set to 1000. For each iteration, the interaction was compared with the counted cells and an interaction score and p-value were obtained. The results, represented in heatmaps, correspond to the significant values at the alpha 1‰ risk.

## Data availability statement

The raw data supporting the conclusions of this article will be made available by the authors, without undue reservation.

## Ethics statement

The animal study was reviewed and approved by Comité d’éthique pour l’expérimentation animale CEEA-LR N°36. Housing and mouse colony management will be performed in there animal facility (IRCM D 34-172-27). Written informed consent was obtained from the owners for the participation of their animals in this study.

## Author contributions

YG performed experiments and wrote a first draft of the manuscript. L-AC processed and analyzed the data and developed analytic tools for data analysis. A-SD contributed to the experimental design and conduction. JP and NP provided FFPE blocks and tissue expertise. NB and H-AM conceived and supervised the study, interpreted results and revised the manuscript. YG and L-AC are first authors and NB and H-AM are senior authors All authors contributed to the article and approved the submitted version.

## Supplementary material

Supplementary Figure 1 Assessment by immunofluorescence analysis of antigen retrieval compatibility of unconjugated antibodies. 1x and 3x (yellow squares) magnified images of each unconjugated antibody on relevant tissues (spleen, lymph nodes, lung, liver, MCA-205 cell tumor and intestine) using the same antigen retrieval conditions (EDTA, pH 9, 96˚C, 30 min) by immunofluorescence.

Supplementary Figure 2 Single-antibody signal by imaging mass cytometry. 1x and 3x (yellow square) magnified images of representative metal-tagged antibody signals in the relevant mouse FFPE tissues: LYVE-1 and pan-actin in lung, PNAd and GATA-3 in lymph node, and E-cadherin, β-catenin, pan-cytokeratin and RORγt in intestine sections. All the other markers were tested with mouse FFPE spleen sections.

Supplementary Figure 3 CD8 staining in the intestinal tumors and adjacent healthy tissue. Yellow lines delimit the tumors according the pathologist’s annotation. CD8 expression (yellow) is homogeneously distributed in the adjacent healthy tissue, but is absent in the tumors. Nuclei are in blue.

Supplementary Figure 4 Segmentation optimization. **(A)** Segmentation masks for the WT intestine tissue section. The left column represents the result of the conventional segmentation pipeline (see Methods). Cell boundaries are expanded. Each cell is in contact with its neighboring cells. The right column represents the result obtained with the optimized pipeline in which cell boundaries are reduced. The zoom in the Peyer’s patch shows that cells are individualized. **(B)** Crop images of the stack of the cell segmentation and the image. **(C)** Manual gating of CD3- and B220-expressing cells (i.e. aberrant phenotype). Both methods lead to the same number of cells. The optimized method reduced by 2-fold the rate of aberrant phenotypes.

## References

[1] Bruni D Angell HK Galon Jérôme . The immune contexture and immunoscore in cancer prognosis and therapeutic efficacy. Nat Rev Cancer (2020) 20(11):662–805. doi: 10.1038/s41568-020-0285-7 32753728

[2] Angell HK Bruni D Barrett JC Herbst R Galon Jérôme . The immunoscore: Colon cancer and beyond. Clin Cancer Res (2020) 26(2):332–95. doi: 10.1158/1078-0432.CCR-18-1851 31413009

[3] Cabrita R Lauss M Sanna A Donia M Skaarup Larsen M Mitra S . Tertiary lymphoid structures improve immunotherapy and survival in melanoma. Nature (2020) 577(7791):561–65. doi: 10.1038/s41586-019-1914-8 31942071

[4] Helmink BA Reddy SM Gao J Zhang S Basar R Thakur R . B cells and tertiary lymphoid structures promote immunotherapy response. Nature (2020) 577(7791):549–55. doi: 10.1038/s41586-019-1922-8 PMC8762581 31942075

[5] Italiano A Bessede A Pulido M Bompas E Piperno-Neumann S Chevreau C . ‘Pembrolizumab in soft-tissue sarcomas with tertiary lymphoid structures: A phase 2 PEMBROSARC trial cohort’. Nat Med (2022) 28(6):1199–206. doi: 10.1038/s41591-022-01821-3 35618839

[6] Fridman WH Meylan M Petitprez F Sun C-M Italiano A Sautès-Fridman C . B cells and tertiary lymphoid structures as determinants of tumour immune contexture and clinical outcome. Nat Rev Clin Oncol (2022) 19(7):441–575. doi: 10.1038/s41571-022-00619-z 35365796

[7] Tsang S In Hassan AA To SKY Wong AST . Experimental models for ovarian cancer research. Exp Cell Res (2022) 416(1):1131505. doi: 10.1016/j.yexcr.2022.113150 35405118

[8] Proetzel G Wiles MV Roopenian DC . Genetically engineered humanized mouse models for preclinical antibody studies. BioDrugs (2014) 28(2):171–805. doi: 10.1007/s40259-013-0071-0 24150980 PMC4414315

[9] Zakarya R Howell VM Colvin EK . Modelling epithelial ovarian cancer in mice: Classical and emerging approaches. Int J Mol Sci (2020) 21(13):48065. doi: 10.3390/ijms21134806 PMC7370285 32645943

[10] Gu Yu Bui T Muller WJ . Exploiting mouse models to recapitulate clinical tumor dormancy and recurrence in breast cancer. Endocrinology (2022) 163(6):bqac055. doi: 10.1210/endocr/bqac055 35560214

[11] Gil Del Alcazar CR Trinh A Alečković Maša Rojas Jimenez E Harper NW Oliphant MUJ . Insights into immune escape during tumor evolution and response to immunotherapy using a rat model of breast cancer. Cancer Immunol Res (2022) 10(6):680–697. doi: 10.1158/2326-6066.CIR-21-0804 PMC9177779 35446942

[12] Freeman GJ Long AJ Iwai Y Bourque K Chernova T Nishimura H . Engagement of the PD-1 immunoinhibitory receptor by a novel B7 family member leads to negative regulation of lymphocyte activation. J Exp Med (2000) 192(7):1027–34. doi: 10.1084/jem.192.7.1027 PMC2193311 11015443

[13] Leach DR Krummel MF Allison JP . Enhancement of antitumor immunity by CTLA-4 blockade. Sci (New York N.Y.) (1996) 271(5256):1734–36. doi: 10.1126/science.271.5256.1734 8596936

[14] Dong Y Sun Q Zhang X . PD-1 and its ligands are important immune checkpoints in cancer. Oncotarget (2017) 8(2):2171–865. doi: 10.18632/oncotarget.13895 PMC5356790 27974689

[15] Hegde PS Chen DS . Top 10 challenges in cancer immunotherapy. Immunity (2020) 52(1):17–355. doi: 10.1016/j.immuni.2019.12.011 31940268

[16] Robinson BWS Redwood AJ Creaney J . How our continuing studies of the pre-clinical inbred mouse models of mesothelioma have influenced the development of new therapies. Front Pharmacol (2022) 13:858557(March). doi: 10.3389/fphar.2022.858557 35431929 PMC9008447

[17] Le Rochais M Hemon P Pers J-O Uguen A . Application of high-throughput imaging mass cytometry Hyperion in cancer research. Front Immunol (2022) 13:859414(March). doi: 10.3389/fimmu.2022.859414 35432353 PMC9009368

[18] Niewold P Ijsselsteijn ME Verreck FAW Ottenhoff THM Joosten SA . An imaging mass cytometry immunophenotyping panel for non-human primate tissues. Front Immunol (2022) 13:915157(July). doi: 10.3389/fimmu.2022.915157 35911721 PMC9334813

[19] Ijsselsteijn ME van der Breggen R Sarasqueta AF Koning F de Miranda NFCC . A 40-marker panel for high dimensional characterization of cancer immune microenvironments by imaging mass cytometry. Front Immunol (2019) 10:2534(October). doi: 10.3389/fimmu.2019.02534 31736961 PMC6830340

[20] Guo N van Unen V Ijsselsteijn ME Ouboter LF van der Meulen AE Chuva de Sousa Lopes SM . A 34-marker panel for imaging mass cytometric analysis of human snap-frozen tissue. Front Immunol (2020) 11:1466(July). doi: 10.3389/fimmu.2020.01466 32765508 PMC7381123

[21] Elaldi R Hemon P Petti L Cosson E Desrues B Sudaka A . High dimensional imaging mass cytometry panel to visualize the tumor immune microenvironment contexture. Front Immunol (2021) 12:666233(April). doi: 10.3389/fimmu.2021.666233 33936105 PMC8085494

[22] Perrot I Michaud H-A Giraudon-Paoli M Augier Séverine Docquier Aurélie Gros L . Blocking antibodies targeting the CD39/CD73 immunosuppressive pathway unleash immune responses in combination cancer therapies. Cell Rep (2019) 27(8):2411–25.e9. doi: 10.1016/j.celrep.2019.04.091 31116985

[23] Porgador A Feldman M Eisenbach L . H-2K ^b^ TRANSFECTION OF B16 MELANOMA CELLS RESULTS IN REDUCED TUMOURIGENICITY AND METASTATIC COMPETENCE. Eur J Immunogenetics (1989) 16(4–5):291–303. doi: 10.1111/j.1744-313X.1989.tb00475.x 2639904

[24] Bertrand F Montfort A Marcheteau E Imbert C Gilhodes J Filleron T . TNFα blockade overcomes resistance to anti-PD-1 in experimental melanoma. Nat Commun (2017) 8(1):2256. doi: 10.1038/s41467-017-02358-7 29273790 PMC5741628

[25] Montfort A Bertrand F Rochotte J Gilhodes J Filleron T Milhès J . Neutral sphingomyelinase 2 heightens anti-melanoma immune responses and anti-PD-1 therapy efficacy. Cancer Immunol Res (2021) 9(5):568–82. doi: 10.1158/2326-6066.CIR-20-0342 PMC9631340 33727246

[26] Colnot S Niwa-Kawakita M Hamard G Godard Cécile Le Plenier S Houbron C . Colorectal cancers in a new mouse model of familial adenomatous polyposis: Influence of genetic and environmental modifiers. Lab Invest (2004) 84(12):1619–305. doi: 10.1038/labinvest.3700180 15502862

[27] Garrido-Mesa N Algieri F Rodríguez Nogales A Gálvez J . Functional plasticity of Th17 cells: Implications in gastrointestinal tract function. Int Rev Immunol (2013) 32(5–6):493–510. doi: 10.3109/08830185.2013.834899 24040751

[28] Luu M Steinhoff U Visekruna A . Functional heterogeneity of gut-resident regulatory T cells. Clin Trans Immunol (2017) 6(9)e156. doi: 10.1038/cti.2017.39 PMC5628268 28983404

[29] Jackson HW Fischer JR Zanotelli VRT Ali HR Mechera R Soysal SD . The single-cell pathology landscape of breast cancer. Nature (2020) 578(7796):615–20. doi: 10.1038/s41586-019-1876-x 31959985

[30] Guéry L Hugues Stéphanie . Th17 cell plasticity and functions in cancer immunity. BioMed Res Int (2015) 2015:314620. doi: 10.1155/2015/314620 26583099 PMC4637016

[31] Chen W Jin W Hardegen N Lei K-J Li Li Marinos N . Conversion of peripheral CD4+CD25- naive T cells to CD4+CD25+ regulatory T cells by TGF-beta induction of transcription factor Foxp3. J Exp Med (2003) 198(12):1875–865. doi: 10.1084/jem.20030152 PMC2194145 14676299

[32] Vitale I Shema E Loi S Galluzzi L . Intratumoral heterogeneity in cancer progression and response to immunotherapy. Nat Med (2021) 27(2):212–45. doi: 10.1038/s41591-021-01233-9 33574607

[33] Goltsev Y Samusik N Kennedy-Darling J Bhate S Hale M Vazquez G . Deep profiling of mouse splenic architecture with CODEX multiplexed imaging’. Cell (2018) 174(4):968–9815.e15. doi: 10.1016/j.cell.2018.07.010 30078711 PMC6086938

[34] Kinkhabwala A Herbel C Pankratz J Yushchenko DA Rüberg S Praveen P . MACSima imaging cyclic staining (MICS) technology reveals combinatorial target pairs for CAR T cell treatment of solid tumors. Sci Rep (2022) 12(1):1911. doi: 10.1038/s41598-022-05841-4 35115587 PMC8813936

[35] Liu CC McCaffrey EF Greenwald NF Soon E Risom T Vijayaragavan K . Multiplexed ion beam imaging: Insights into pathobiology. Annu Rev Pathology: Mech Dis (2022) 17(1):403–23. doi: 10.1146/annurev-pathmechdis-030321-091459 34752710

[36] Devine RD Behbehani GK . Mass cytometry, imaging mass cytometry, and multiplexed ion beam imaging use in a clinical setting. Clinics Lab Med (2021) 41(2):297–3085. doi: 10.1016/j.cll.2021.03.008 34020765

[37] Strittmatter N Richards FM Race AM Ling S Sutton D Nilsson A . Method to visualize the intratumor distribution and impact of gemcitabine in pancreatic ductal adenocarcinoma by multimodal imaging. Analytical Chem (2022) 94(3):1795–803. doi: 10.1021/acs.analchem.1c04579 35005896

[38] Strittmatter N Moss JI Race AM Sutton D Canales JR Ling S . Multi-modal molecular imaging maps the correlation between tumor microenvironments and nanomedicine distribution. Theranostics (2022) 12(5):2162–74. doi: 10.7150/thno.68000 PMC8899579 35265205

[39] Peran I Dakshanamurthy S McCoy MD Mavropoulos A Allo B Sebastian A . Cadherin 11 promotes immunosuppression and extracellular matrix deposition to support growth of pancreatic tumors and resistance to gemcitabine in mice. Gastroenterology (2021) 160(4):1359–72.e13. doi: 10.1053/j.gastro.2020.11.044 33307028 PMC7956114

[40] Liu H-C Viswanath DI Pesaresi F Xu Y Zhang L Di Trani N . Potentiating antitumor efficacy through radiation and sustained intratumoral delivery of anti-CD40 and anti-PDL1. Int J Radiat OncologyBiologyPhysics (2021) 110(2):492–506. doi: 10.1016/j.ijrobp.2020.07.2326 PMC8547413 32768562

[41] Melin N Yarahmadov T Daniel Sanchez-Taltavull Birrer FE Brodie TM Petit Benoît . A new mouse model of radiation-induced liver disease reveals mitochondrial dysfunction as an underlying fibrotic stimulus. JHEP Rep (2022) 4(7):100508. doi: 10.1016/j.jhepr.2022.100508 35712694 PMC9192810

[42] Gheiratmand L Brown DJ Sandkuijl D Loboda A Jester JV . Immuno tomography (IT) and imaging mass cytometry (IMC) for constructing spatially resolved, multiplexed 3D IMC data sets. Ocular Surface (2022) 25(July):49–54. doi: 10.1016/j.jtos.2022.04.008 35489589 PMC10411503

[43] Lotsberg ML Røsland GV Rayford AJ Dyrstad SE Ekanger CT Lu N . Intrinsic differences in spatiotemporal organization and stromal cell interactions between isogenic lung cancer cells of epithelial and mesenchymal phenotypes revealed by high-dimensional single-cell analysis of heterotypic 3D spheroid models. Front Oncol (2022) 12:818437(April). doi: 10.3389/fonc.2022.818437 35530312 PMC9076321

[44] Zanotelli VRT Bodenmiller B . ImcSegmentationPipeline: A pixelclassification based multiplexed image segmentation pipeline. Zenodo (2017). doi: 10.5281/ZENODO.3841961

[45] Andrzej Oleś GP . ‘EBImage—an R package for image processing with applications to cellular phenotypes’. Bioinformatics (2017) 26(7):979–1. doi: 10.1093/bioinformatics/btq046 PMC2844988 20338898

[46] Ellis B Hahne F Ellis B Haaland P . FlowCore: Data structures package for flow cytometry data. BMC Bioinformatics (2007) 10:106. doi: 10.1186/1471-2105-10-106 PMC2684747 19358741

[47] Raivo K . Pheatmap: Pretty heatmaps. r package version 1.0.12. (2019).

[48] Lê S Josse J Husson F . FactoMineR: An r package for multivariate analysis. J Stat Software (2008) 25(1):p1–18. hal-00359835.

[49] Wickham H . Ggplot2: Elegant graphics for data analysis. 2nd ed Vol. 2016. . Cham: Springer International Publishing : Imprint: Springer (2016). doi: 10.1007/978-3-319-24277-4

[50] Windhager J Bodenmiller B Eling N . An end-to-End workflow for multiplexed image processing and analysis. bioRxiv (2021). doi: 10.1101/2021.11.12.468357 37816904

[51] Eling N Damond N Hoch T Bodenmiller B . *Cytomapper* : An R/Bioconductor package for visualization of highly multiplexed imaging data. Bioinformatics (2021) 36(24):5706–85. doi: 10.1093/bioinformatics/btaa1061 PMC8023672 33367748

