# Supplementary figures and images for: A 31-plex panel for high-dimensional single-cell analysis of murine preclinical models of solid tumors by imaging mass cytometry
Source: Front Immunol. 2023 Jan 19;13:1011617. doi: 10.3389/fimmu.2022.1011617 (PMC9893499; doi:10.3389/fimmu.2022.1011617)

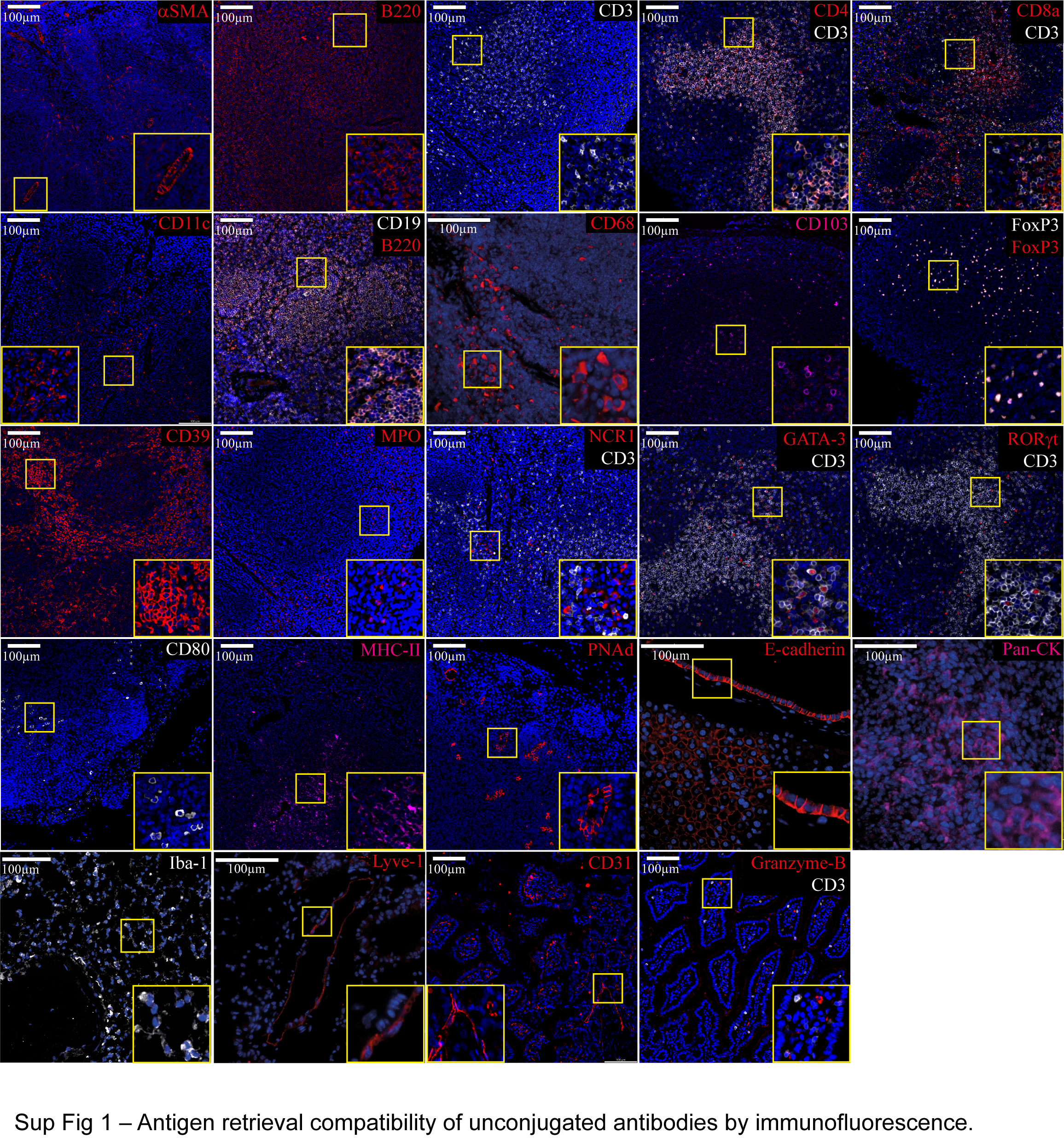

Supplement: Supplementary Figure 1 — Assessment by immunofluorescence analysis of antigen retrieval compatibility of unconjugated antibodies. 1x and 3x (yellow squares) magnified images of each unconjugated antibody on relevant tissues (spleen, lymph nodes, lung, liver, MCA-205 cell tumor and intestine) using the same antigen retrieval conditions (EDTA, pH 9, 96˚C, 30 min) by immunofluorescence. [file Image_1.tif]

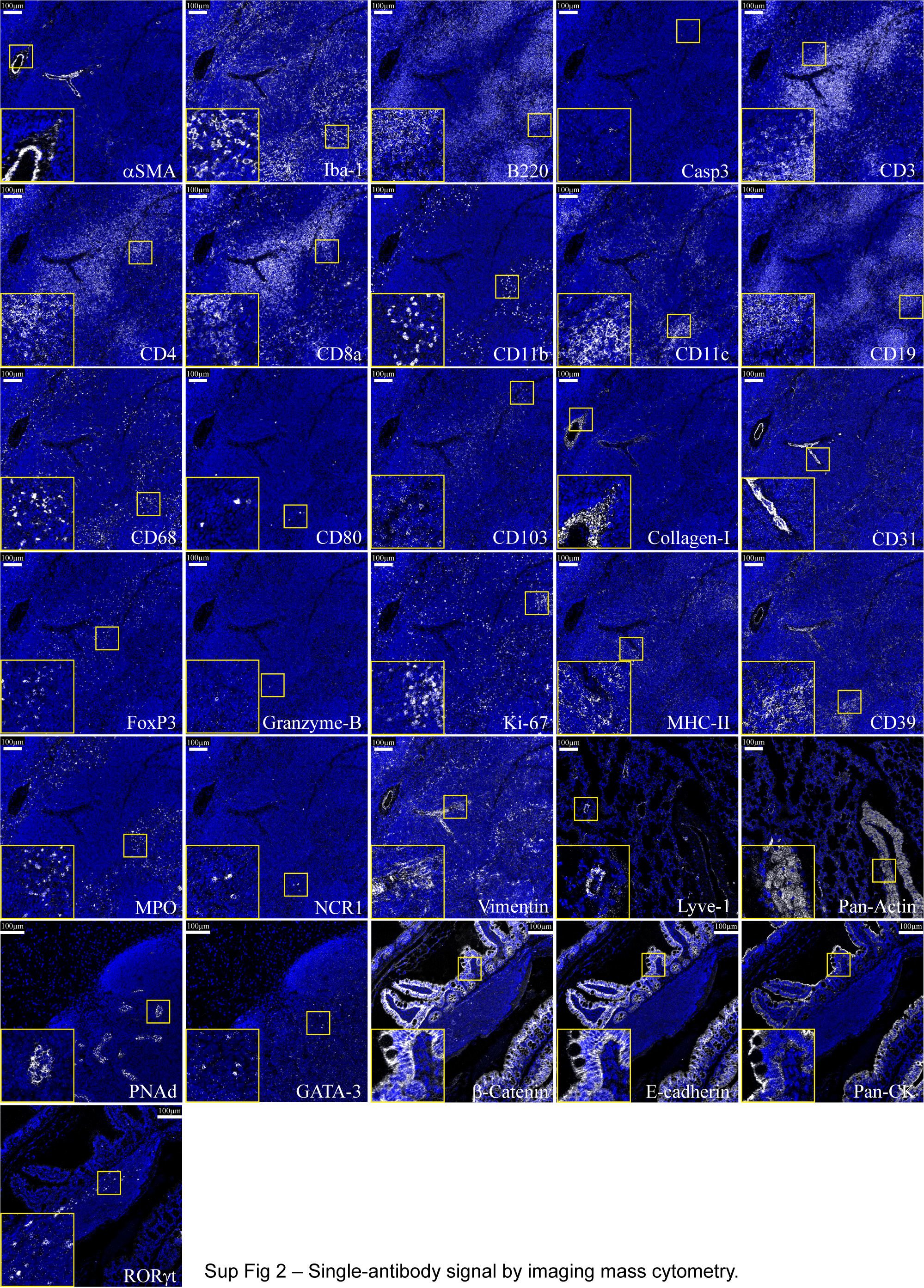

Supplement: Supplementary Figure 2 — Single-antibody signal by imaging mass cytometry. 1x and 3x (yellow square) magnified images of representative metal-tagged antibody signals in the relevant mouse FFPE tissues: LYVE-1 and pan-actin in lung, PNAd and GATA-3 in lymph node, and E-cadherin, β-catenin, pan-cytokeratin and RORγt in intestine sections. All the other markers were tested with mouse FFPE spleen sections. [file Image_2.tif]

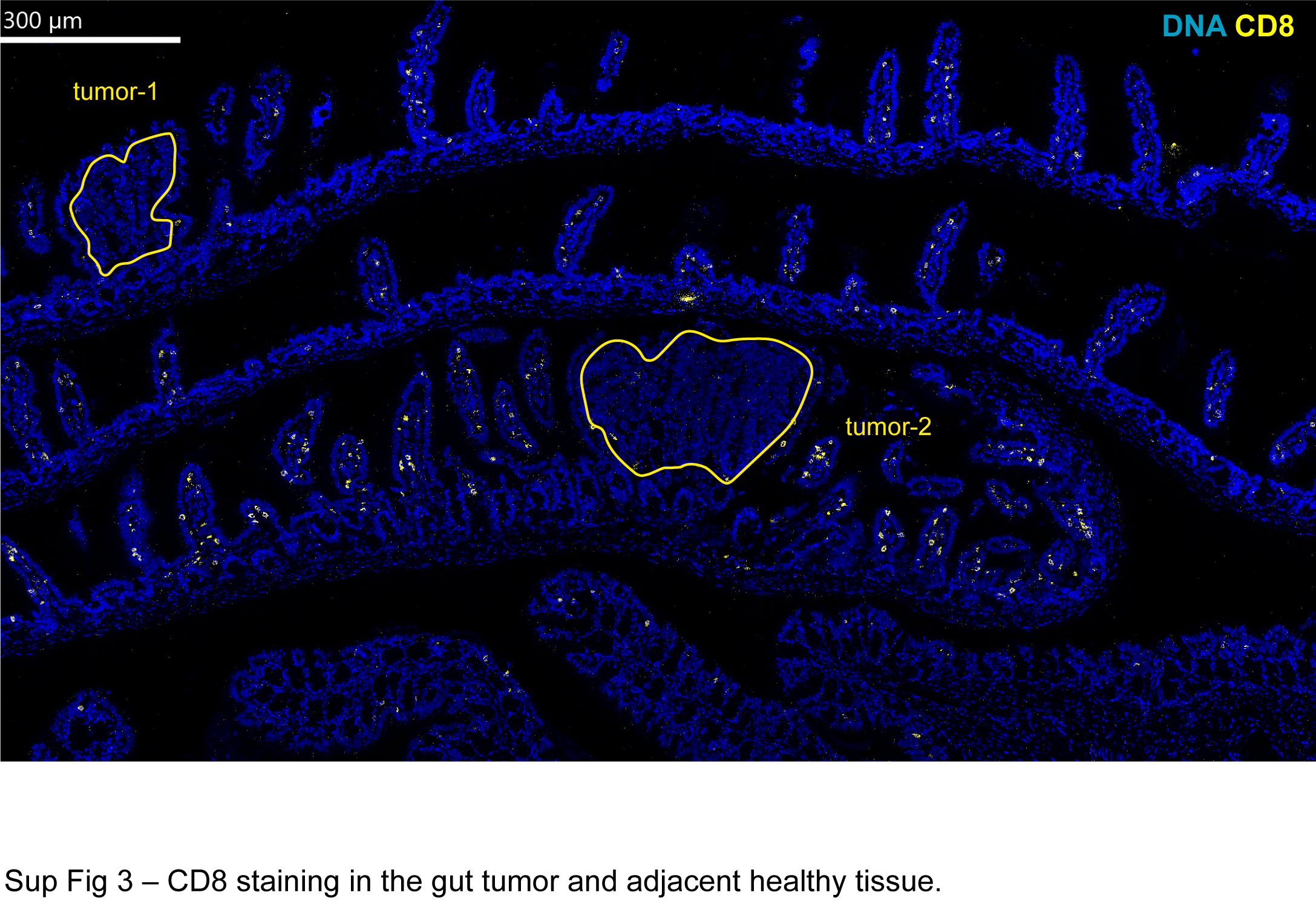

Supplement: Supplementary Figure 3 — CD8 staining in the intestinal tumors and adjacent healthy tissue. Yellow lines delimit the tumors according the pathologist’s annotation. CD8 expression (yellow) is homogeneously distributed in the adjacent healthy tissue, but is absent in the tumors. Nuclei are in blue. [file Image_3.tif]

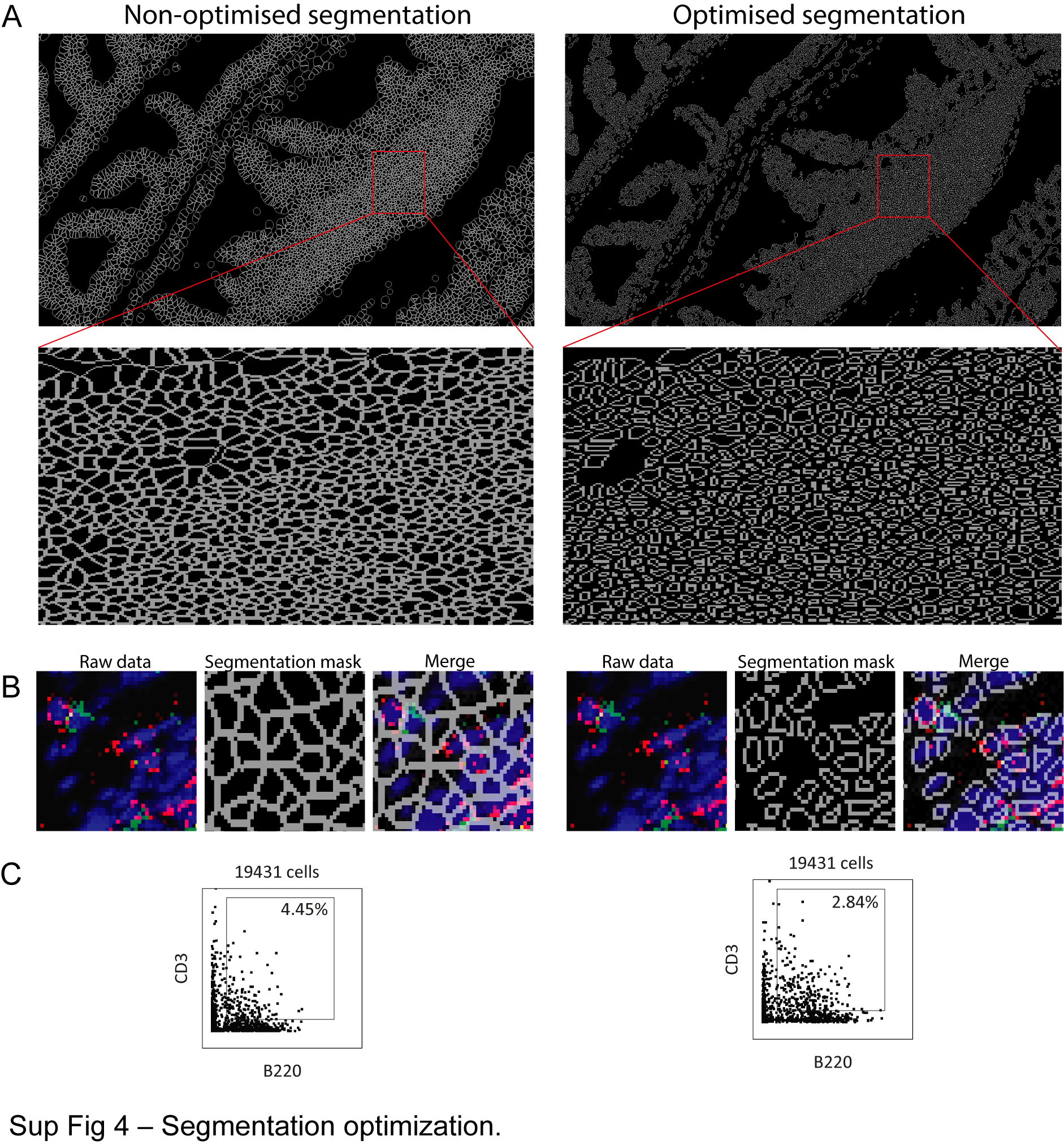

Supplement: Supplementary Figure 4 — Segmentation optimization. (A) Segmentation masks for the WT intestine tissue section. The left column represents the result of the conventional segmentation pipeline (see Methods). Cell boundaries are expanded. Each cell is in contact with its neighboring cells. The right column represents the result obtained with the optimized pipeline in which cell boundaries are reduced. The zoom in the Peyer’s patch shows that cells are individualized. (B) Crop images of the stack of the cell segmentation and the image. (C) Manual gating of CD3- and B220-expressing cells (i.e. aberrant phenotype). Both methods lead to the same number of cells. The optimized method reduced by 2-fold the rate of aberrant phenotypes. [file Image_4.tif]
